# Multi-Locus Sequence Analysis Indicates Potential Cryptic Speciation in the Chigger Mite *Neoschoengastia gallinarum* (Hatori, 1920) Parasitising Birds in Asia

**DOI:** 10.3390/ani14060980

**Published:** 2024-03-21

**Authors:** Praveena Rajasegaran, Sirikamon Koosakulnirand, Kim-Kee Tan, Jing Jing Khoo, Youseuf Suliman, Mohammad Saiful Mansor, Mohd K. S. Ahmad Khusaini, Sazaly AbuBakar, Kittipong Chaisiri, Serge Morand, Zubaidah Ya’cob, Benjamin L. Makepeace

**Affiliations:** 1 Tropical Infectious Diseases Research and Education Centre (TIDREC), Higher Institution Centre of Excellence (HICoE), Universiti Malaya, Kuala Lumpur 50603, Malaysia; praveenarasi94@gmail.com (P.R.); kimkee@um.edu.my (K.-K.T.); sazaly@um.edu.my (S.A.); zyacob@um.edu.my (Z.Y.); 2 Institute for Advanced Studies, Universiti Malaya, Kuala Lumpur 50603, Malaysia; 3 Institute of Infection, Veterinary & Ecological Sciences, University of Liverpool, Liverpool L3 5RF, UK; s.koosakulnirand@liverpool.ac.uk (S.K.); jing.jing.khoo@liverpool.ac.uk (J.J.K.); y.m.suliman@liverpool.ac.uk (Y.S.); 4 Department of Microbiology & Immunology, Faculty of Tropical Medicine, Mahidol University, Bangkok 10400, Thailand; 5 Department of Biological Sciences and Biotechnology, Faculty of Science & Technology, Universiti Kebangsaan Malaysia, Bangi 43600, Malaysia; msaifulmansor@ukm.edu.my; 6 Wildlife Conservation Division, Department of Wildlife and National Parks Peninsular Malaysia, Ministry of Natural Resources, Environment and Climate Change, Kuala Lumpur 56100, Malaysia; khusainikharip@gmail.com; 7 Department of Helminthology, Faculty of Tropical Medicine, Mahidol University, Bangkok 10400, Thailand; kittipong.chaisiri@gmail.com; 8 IRL HealthDEEP, CNRS-Kasetsart University-Mahidol University, Bangkok 10900, Thailand; serge.morand@umontpellier.fr

**Keywords:** Galliformes, trombiculid, molecular barcoding, trombiculiasis, chickens

## Abstract

**Simple Summary:**

The chigger mite *Neoschoengastia gallinarum* (Hatori, 1920) is a parasite that feeds on the skin tissue of birds across multiple countries in Southeast and East Asia. In domestic chickens, heavy infestations with this mite can lead to skin irritation and damage to the carcass, reducing economic value. In this study, we collected *N. gallinarum* samples from wild birds of conservation concern and domestic chickens in Peninsular Malaysia and Thailand. Sequence analyses of three genes from the mites were compared across four Malaysian populations, one Thai population, and previously published sequences from southeastern China. A variety of methods were applied to classify these sequences and determine the extent of interbreeding between populations. These methods agreed in identifying three clusters of sequences by country of origin, although there was partial overlap between Thailand and China. The populations from Malaysia and Thailand appear to be reproductively isolated from one another and may represent distinct species with almost identical morphological features, except for leg length. Further studies are required to determine if these genetic dissimilarities are accompanied by distinct ecological, behavioural, or pathological differences in *N. gallinarum* in different regions of Asia.

**Abstract:**

*Neoschoengastia gallinarum* is widely distributed in Asia, preferentially parasitising birds, and heavy infestations have clinical impacts on domestic fowl. In common with other trombiculid mites, the genetic diversity and potential variation in host preferences or pathology induced by *N. gallinarum* are poorly understood. This study aimed to unravel the geographical variation and population structure of *N. gallinarum* collected from galliform birds in Peninsular Malaysia and Thailand by inference from concatenated mitochondrial-encoded cytochrome c oxidase subunit I (COI), and nuclear-encoded internal transcribed spacer 2 (ITS2) and 18S ribosomal DNA gene sequences, including a comparison with previously published data from southeastern China. Our multi-locus sequence analysis revealed three monophyletic clades comprising (A) specimens from Peninsular Malaysia, (B) the samples from Thailand together with a minority of Chinese sequences, and (C) the majority of sequences from China. Similarly, most species delimitation approaches divided the specimens into three operational taxonomic units. Analysis of molecular variance revealed 96.41% genetic divergence between Malaysian and Thai populations, further supported by the absence of gene flow (N*m* = 0.01). In conclusion, despite the two countries sharing a land border, populations of *N. gallinarum* from Peninsular Malaysia and Thailand appear to be genetically segregated and may represent distinct cryptic species.

## 1. Introduction

Trombiculid mite larvae or “chiggers” (Actinotrichida: Trombiculidae) are globally distributed etiological agents of trombiculiasis, a form of dermatitis resulting from their bites [1,2,3]. Trombiculiasis can affect a wide range of wild and domestic terrestrial vertebrate hosts, including humans, in which the condition is sometimes referred to as “scrub itch”. During feeding, a straw-like structure called the stylostome is formed from compounds in the chiggers’ saliva reacting with the host’s tissues, creating a tube that extends from their mouthparts. The saliva is also thought to contain lytic enzymes and anticoagulants, which facilitate the imbibement of tissue fluid and liquified skin cells [4,5]. Hypersensitivity reactions to mite allergens may then proceed at the bite site [6,7], especially in atopic hosts, leading to potentially severe dermatitis in a variety of host species [8,9,10]. Crater-like pits and nodular lesions caused by chigger bites have been reported in mammals infested with chigger species from the genera *Euschoengastia* Ewing, 1938, *Gahrliepia* Oudemans, 1912, *Hyponeocula* Vercammen-Grandjean, 1960, and *Schoutedenichia* Jadin and Vercammen-Grandjean, 1954 [11,12,13,14,15,16]. Additionally, several studies have described chigger-induced skin lesions in both domestic and wild birds, sometimes accompanied by poor body condition or even mortality [2,17,18]. Importantly, some chigger species have a major clinical impact on humans as vectors of scrub typhus, a potentially fatal zoonosis caused by *Orientia* spp. bacteria [19]. However, the chigger genus primarily responsible for *Orientia* transmission to humans (*Leptotrombidium*) is not a major cause of scrub itch compared with members of the genera *Eutrombicula* Ewing, 1938, *Schoengastia* Oudemans, 1910, and *Neotrombicula* Hirst, 1925 [20].

The genus *Neoschoengastia* Ewing, 1929 has a global distribution with over 70 recorded species, most of which have a marked predilection for domestic or wild avian hosts [21,22,23]. While certain *Neoschoengastia* spp. have been recorded on mammalian hosts such as rodents and ungulates [24,25], they are not a recognised cause of scrub itch in humans. However, *Neoschoengastia* spp. are significant pests of domestic fowl, especially for turkeys in North America [*Neoschoengastia americana* (Hirst, 1921)], common pheasants in Japan (*Neoschoengastia shiraii* Sasa and Sato, 1953), and chickens in East and Southeast Asia (*Neoschoengastia gallinarum*) [26,27,28]. Recently, *N. gallinarum* was recorded for the first time in Thailand (parasitising domestic chickens), as well as being found in abundance on wild Galliformes [*Gallus gallus* (Linnaeus, 1758)*, Lophura rufa* (Raffles, 1822), *Polyplectron inopinatum* (Rothschild, 1903), and *Polyplectron malacense* (Scopoli, 1786)] in Peninsular Malaysia [29]. China, Taiwan, and Vietnam are also included in this species’ range [23], which is widespread and greatly reduces the economic value of poultry due to damage to the carcass [28]. However, there is a gap in knowledge concerning the genetic diversity and potential variation in host preferences or pathology induced by *N. gallinarum* across its endemic regions of East and Southeast Asia.

The use of molecular approaches for chigger species discrimination has been very limited until recently. However, the application of molecular barcoding based on the mitochondrially encoded cytochrome c oxidase subunit I (COI) gene [or occasionally the nuclear-encoded internal transcribed spacer 2 (ITS2) region] is becoming more widespread in the chigger field, with several studies from Asia and Europe using this approach for *Leptotrombidium* spp. and a number of other genera [30,31,32,33,34,35]. Although such analyses should be interpreted with caution since they are based on a single gene, they indicate that some chigger species with identical barcodes can display morphological plasticity on different hosts, whereas other species exhibit polymorphisms in the COI region without accompanying morphological variation. Notably, *N. gallinarum* is the only chigger species in which more than two genes have been applied in population genetic studies. Zhou et al. [30] used portions of the 18S and 28S rRNA genes, the complete ITS2 region, and a COI fragment to study the population structure of *N. gallinarum* in the Fujian and Guangdong provinces of southeastern China. They reported that two genotypes of COI were present, which were not linked to geographical location or morphological variation, and the relatively conserved nuclear markers did not show polymorphisms associated with the COI genotypes. They concluded that COI is useful for both interspecies and intraspecies phylogenetic analyses and the discovery of new genotypes. Meanwhile, the ITS2 and 18S rDNA genes are relatively conserved and more suitable for analysing interspecies variation and species-level identification. Here, with the aim of unravelling the geographical variation and population structure of *N. gallinarum* in Peninsular Malaysia and Thailand, we performed multi-locus sequence analyses using concatenated COI, ITS2, and 18S rRNA genes. Moreover, we applied comparative analyses with published sequences available for the Chinese populations to determine whether *N. gallinarum* displays panmixia across Asia or forms reproductively isolated populations. We present evidence suggesting that the *N. gallinarum* populations of Peninsular Malaysia, Thailand, and southeastern China constitute at least two and possibly three cryptic species.

## 2. Material and Methods

### 2.1. Study Sites and Chigger Collections

**Figure 1:**
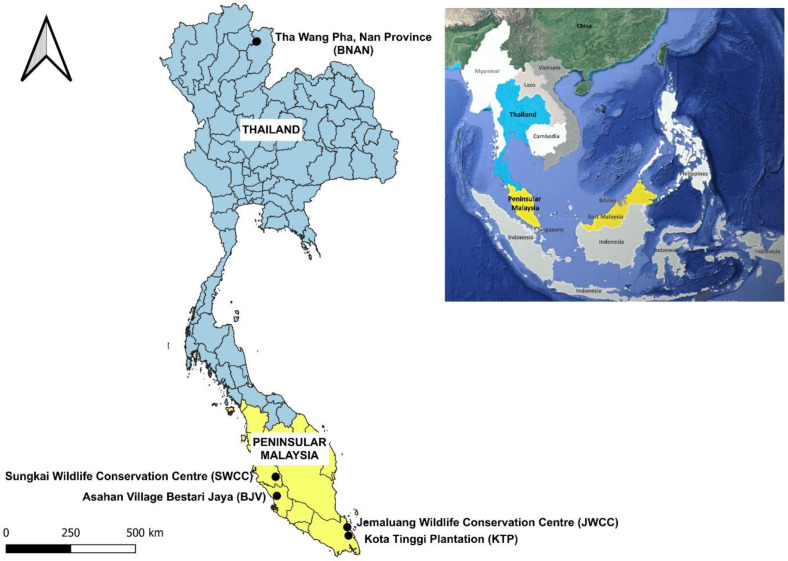
Map illustrating the five study sites in Peninsular Malaysia and Thailand. The inset map displays the Southeast Asian region. The main map shows the sampling localities within Malaysia [Sungkai Wildlife Conservation Centre, Perak (SWCC); Asahan Village Bestari Jaya, Selangor (BJV); Jemaluang Wildlife Conservation Centre, Johor (JWCC); Kota Tinggi Plantation, Johor (KTP)] and Saen Thong subdistrict, Tha Wang Pha district, Nan province, Thailand (BNAN).

**Figure 2:**
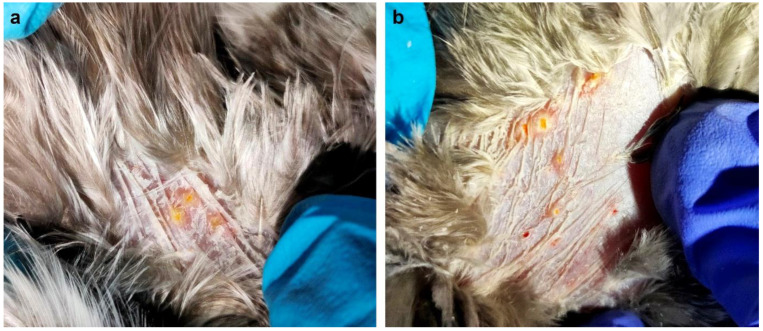
Multifocal coalescing pattern of chigger infestation on the dermal surface of a Malayan crested fireback (*Lophura rufa*), specifically on the (**a**) thigh and (**b**) breast areas.

The sampling effort for the collection of *N. gallinarum* [36] from infested galliform birds was conducted at four sites in Peninsular Malaysia [Sungkai Wildlife Conservation Centre, Perak (code SWCC)—January 2021 and March 2021; Asahan Village Bestari Jaya, Selangor (BJV)—April 2021; Jemaluang Wildlife Conservation Centre, Johor (JWCC)—February 2022; Kota Tinggi Plantation, Johor (KTP)—June 2022]. Only a single site in Thailand was sampled [Saen Thong subdistrict, comprising two villages—Ban Huay Muang and Ban Santisuk—in Tha Wang Pha district, Nan province (BNAN)] in December 2022, during activities of the One Health Observatory project (ANR FutureHealthSEA) [37] (Figure 1). Details of samples collected from the five sites from each species of host are summarised in Table 1. Chigger mites were removed from predilection sites on the birds’ skin (mainly breast and thigh—see Figure 2) using fine forceps. The recovered chiggers were stored in 70% ethanol at −20 °C. Chiggers from each host were counted and 10% of specimens were selected for mounting in Berlese fluid for species-level identification using an Axio Imager M2 microscope (Zeiss, Oberkochen, Germany) and ZEN 2011 imaging software [31]. These individuals were not used for DNA extraction but were retained as voucher specimens and deposited at the Tick Cell Biobank Asia Outposts Laboratory, Tropical Infectious Diseases Research & Education Centre, Universiti Malaya [29]. The remaining chiggers from each bird host were identified using the autofluorescence method [31] on a GXM-L3201 LED research fluorescence trinocular microscope (GT Vision LTD, Newmarket, UK) with reference to the voucher specimens.

### 2.2. DNA Extraction from Chiggers

Total genomic DNA was extracted from individual chigger mites of *N. gallinarum* using a QIAamp DNA Micro Kit (Qiagen, Redwood City, CA, USA) following the manufacturer’s protocol. Briefly, the chiggers were washed in nuclease-free water for ethanol elimination. Next, chigger samples were digested in 180 µL tissue lysis buffer with 20 µL proteinase K and incubated at 56 °C overnight. The kit manufacturer’s instructions were continued with the DNA recovered in 30 µL elution buffer and stored at −20 °C.

### 2.3. PCR Amplification and Sequencing of PCR Products

Amplifications of the extracted genomic DNA were performed using a universal invertebrate COI (forward–LCO1490: 5′-GGTCAACAAATCATAAAGATATTGG-3′; reverse–HCO2198: 5′-TAAACTTCAGGGTGACCAAAAAATCA-3′) primer pair [38], specific assays targeting ITS2 (forward–5.8S: 5′-CACGCCGAGCACTCGACATT-3′; reverse–28S: 5′-GATCCTTCGCTCGCCGTTACT-3′), 18S ribosomal DNA (18S) (forward–5′-GGCTCATTAAATCAGTTACGGTT-3′; reverse–5′-ATTCCTCGTTCATGGGCAAT-3′) [30], and an ND5 mitochondrial gene fragment (forward–5′-TTTCTGTATTCTGAGCCTTCT-3′; reverse–5′-ATAATAGGGGTTAGCAGAG-3′) [39] of *N. gallinarum*. Polymerase chain reaction (PCR) amplifications were conducted in 25 µL reaction volumes including 2 μL DNA template, 12.5 μL 5X Green DreamTaq Buffer, and 1 μL each primer (final concentration, 0.4 μM) in a 96-well SimpliAmp Thermal Cycler (Applied Biosystems, Inc., Foster City, CA, USA). The amplification profile was as follows: pre-denaturation at 95 °C (2 min), followed by 35 cycles of 95 °C (1 min) for denaturation; 40 °C (1 min) for annealing; 72 °C (1 min and 30 s) for extension; and a final extension at 72 °C (7 min) for COI. For ITS2 and 18S, the programme constituted 94 °C (5 min) for pre-denaturation, followed by 35 cycles of denaturation at 94 °C (30 s); annealing at 55 °C (30 s); extension at 72 °C (30 s); and a final extension at 72 °C (5 min). Lastly, for ND5, the amplification profile begins with pre-denaturation at 94 °C (5 min), followed by 35 cycles of 94 °C (30 s) for denaturation; 54 °C (30 s) for annealing; 72 °C (40 s) for extension; and a final extension at 72 °C (7 min). The amplified PCR products were electrophoresed on a 1.0% agarose gel to determine the product size before submission to Apical Scientific Laboratory Sequencing Company, Selangor, Malaysia, for further purification and Sanger sequencing.

### 2.4. Sequence Alignment

Both forward and reverse sequences of COI, ITS2, and 18S were analysed and edited using BioEdit v7.2.5 [40]. However, we were unable to amplify the ND5 gene fragment of *N. gallinarum* using primers from Tao et al. [39]. All successfully amplified sequences were later aligned using the ClustalX [41] program implemented in BioEdit v7.2.5 [40]. Sequences of COI, ITS2, and 18S of *N. gallinarum* were deposited in the National Center of Biotechnology Information (NCBI) GenBank database under accession numbers OR632279-OR632323, OR636401-OR636445, and OR632359-OR632403, respectively (Table 1).

The aligned COI (551 bp), ITS2 (260 bp), and 18S (729 bp) gene sequences were concatenated using Molecular Evolutionary Genetic Analysis (MEGA) software (version 11.0.11) [42], and the congruency of different partitions among these genes was calculated using a partition homogeneity test of 100 replicates implemented in PAUP 4.0a169 [43]. This generated a *p*-value of 0.87, indicating that the concatenated dataset was congruent between constituent genes. Thus, the 1540 bp concatenated alignment of COI, ITS2, and 18S of *N. gallinarum* was used in the present study.

### 2.5. Phylogenetic Reconstruction and Haplotype Network

The MEGA software (version 11.0.11) [42] was used to run Modeltest to estimate the best evolutionary model of nucleotide substitution for the concatenated sequences. Tamura 3-parameter (T92) with gamma (G) distribution rates showed the lowest Bayesian Information Criterion (BIC) and was chosen to best describe the substitution pattern in the rest of the phylogenetic analysis. Further, MEGA11 was used to compute a pairwise distance using the Kimura 2-parameter (K2P) model [44]. An initial phylogenetic tree was constructed using the Neighbour Joining (NJ) method inferred in MEGA11 with 1000 bootstrap replicates for individual genes of COI, ITS2, 18S, and concatenated datasets. Maximum Likelihood (ML) analysis was also computed on individual genes and concatenated datasets using online phylogeny software, PhyML 3.0, with an automated model selection using BIC [45]. Bayesian inference (BI) analysis was run for the concatenated dataset using MrBayes version 3.2.7 [46]. The Hasegawa–Kishono–Yano substitution model with a gamma shape parameter of 0.109 (HKY + G) was favoured as the best model by jModeltest2 [47] and implemented in the online server CIPRES Science Gateway v3.3 (https://www.phylo.org/, accessed 1 March 2024) [48]. The BI analysis was performed on two million generations of Markov Chain Monte Carlo (MCMC), and the tree was sampled every 100th generation, with the first 10% of trees discarded as burn-in. A total of 10 sequences—8 of *N. gallinarum* (COI–MK423976, MK423977, MK423978; ITS2–MK423979, MK423981, MK643333, MK643334; 18S–MK400440) from the study by Zhou et al. [30] and 3 of *Tetranychus urticae* C. L. Koch, 1836 (Acarina: Trombidiformes; COI-EU345430.1, ITS2-MH919319.1, and 18S-AB926313.1)—were obtained from GenBank and concatenated accordingly. Together with the 45 sequences from Peninsular Malaysia and Thailand, these sequences were selected to study the phylogenetic relationship with *T. urticae* as the outgroup. All trees were visualised in FigTree v1.4.4 and edited in the Interactive Tree of Life (iTOL) [49]. Minimum spanning networks (MSN) [50] among haplotypes were computed using TCS Network [51] and illustrated in PopArt v1.7 [52] to acquire a graphical representation of concatenated COI, ITS2, and 18S data.

### 2.6. Species Delimitation Analyses

Assemble Species by Automatic Partitioning (ASAP) [53], Automatic Barcode Gap Discovery (ABGD) [54], multi-rate Poisson Tree Processes (mPTP) [55], and Generalised Mixed Yule Coalescent (GMYC) [56] were used for species delimitation analyses. Both ASAP and ABGD were performed on a web-based server (ASAP: https://bioinfo.mnhn.fr/abi/public/asap, accessed on 8 January 2024; ABGD: https://bioinfo.mnhn.fr/abi/public/abgd/abgdweb.html, accessed on 8 January 2024) using a Kimura (K80) model with default settings, TS/TV model 2.0 [53,57]. Additionally, for ABGD entity recognition, settings were based on the suggested partition at *P* = 0.01, a relative gap width of 1 and 50 steps, P_min_ = 0.001, P_max_ = 0.1, and Nb bins for distance distribution = 20 [53]. The mPTP delimitation analysis was performed on an mPTP web service available at http://mptp.h-its.org, accessed on 1 March 2024 [55]. To initiate the GMYC species delimitation method for the concatenated dataset, an ultrametric tree was generated using BEAST v2.6.6 [58] to run on the CIPRES Science Gateway v3.3 online portal (https://www.phylo.org/, accessed 1 March 2024) [59]. Preceding this, an XML input file was created using BEAUti v2.6.6 [58] with the best-fitting model, namely (HKY + G) substitution, as determined by jModelTest2 [47]. The Markov Chain Monte Carlo (MCMC) chains were run for 30 million generations, with topologies and parameters logged every 1000 generations. The analysis was then confirmed using Tracer v1.7.1 [60] for an Effective Sampling Size (ESS) of more than 200, demonstrating that the MCMC chains had adequately converged [61]. The output tree was analysed in TreeAnnotator 2.6.6 [58], discarding the initial 10% as burn-in. The subsequent GMYC analysis for the concatenated dataset was conducted in RStudio [62] using R packages v4.3.0, including “ape” [63], “paran” [64], “rncl” [65], and “splits” [66].

### 2.7. Population Genetic and Demographic Analysis

Gene flow was determined by computing the level of population subdivision (*F*_ST_) and the number of migrants (N*m*), also using DnaSP software version 6.12.03 [67]. To resolve the interrelation between geographical distance and genetic differentiation between populations, the Mantel test was conducted in Arlequin version 3.5.2.2 [68] using 1000 permutations [69,70]. Finally, populations were divided into the broad geographical groups of Malaysia and Thailand to study the pattern of genetic structure based on the region of origin, which was examined using an analysis of molecular variance (AMOVA) by estimating the *F*-statistic (Φ_ST_) values with 1000 permutations in Arlequin software 3.5.2.2.

## 3. Results

### 3.1. Identification Confirmation and Sequence Characteristics

**Figure 3:**
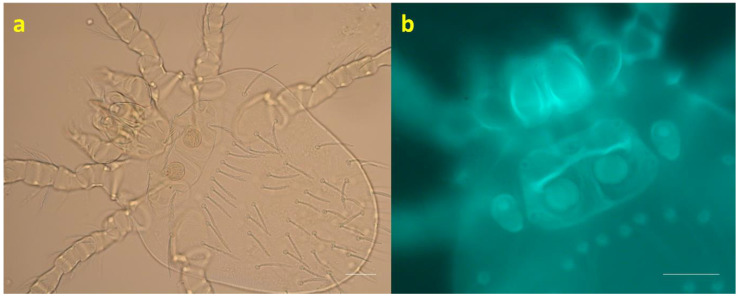
(**a**) Brightfield microscopic view of *N. gallinarum*; (**b**) autofluorescence (AF) imaging of *N. gallinarum* scutum (scale bars, 10 μm). Both images were obtained using a Zeiss Axio Imager M2 microscope and ZEN 2011 imaging software. The host was a Malayan peacock-pheasant (*Polyplectron malacense*).

The trombiculid mites collected from Galliformes were morphologically screened and measured, referring to Domrow and Nadchatram [71], which confirmed their identification as *N. gallinarum* (Figure 3) [36]. No difference in key characteristics was found for this species between Peninsular Malaysia and Thailand except for the total length of legs (Table 2). The diagnostic characters of the *N. gallinarum* mounted for brightfield microscopy were barbed galeal setae, a coxal formula of I.I.I, a palpal setal formula of BBNBB + 7B, and a scutal formula of AL > PL > AM [71], with measurements as shown in Table 2.

Segments of COI, ITS2, and 18S were successfully sequenced and concatenated from 45 individuals of *N. gallinarum* with a final alignment length of 1540 bp. Of these, 1384 were conserved sites, whereas 55 were variable sites (comprising eight singleton variable sites and 47 parsimony-informative sites).

### 3.2. Phylogenetic Reconstruction

**Figure 4:**
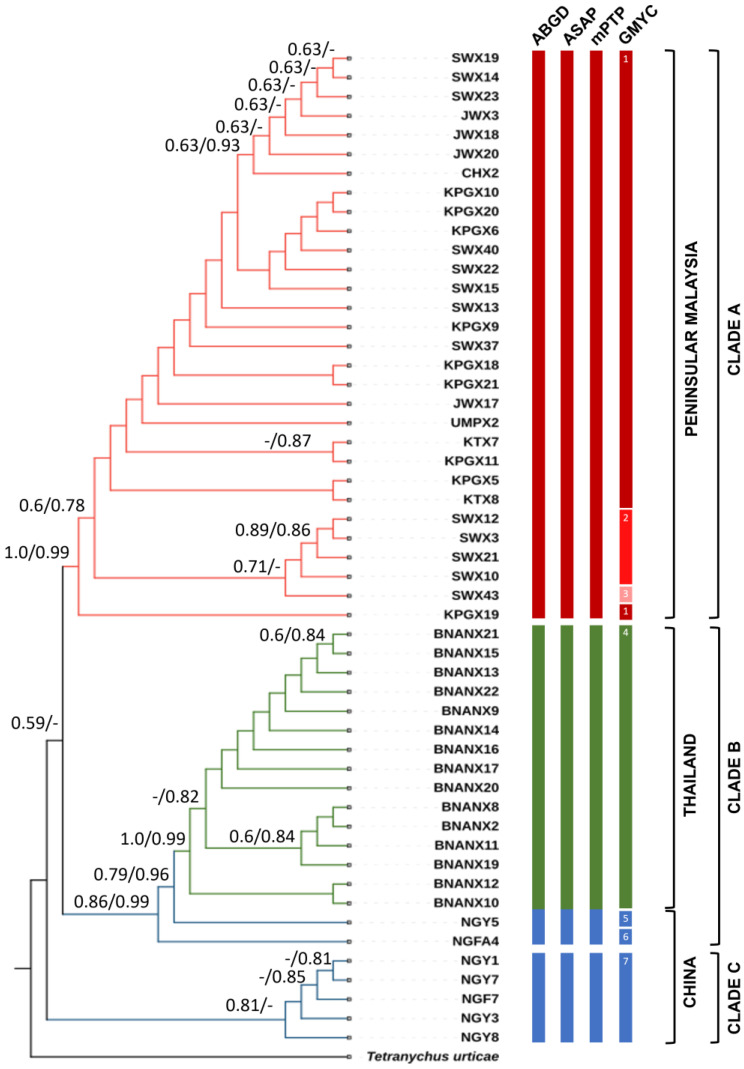
Phylogenetic relationships among *N. gallinarum* populations from Peninsular Malaysia (red), Thailand (green), and China (blue) inferred through Neighbour Joining (NJ) and Maximum Likelihood (ML) analysis based on the concatenated nucleotide sequences of mitochondrial cytochrome c oxidase subunit 1, second internal transcribed spacer, and 18S ribosomal DNA. Bootstrap values (NJ/ML) are shown on the branches. Vertical bars on the right are the results of species delimitation by ABGD, ASAP, mPTP, and GMYC with the population groups indicated to the right. The numbers in the vertical bars of GMYC indicate the OTUs assigned from that analysis.

The phylogenetic analysis of 45 individuals from this study was complemented by including 7 concatenated, published *N. gallinarum* sequences from Zhou et al. [30]. The topology was similar for phylogenetic trees constructed by different methods [i.e., ML or NJ (Figure 4) and BI (Figure S1)]. The tree was divided into three main clades, of which the Malaysian clade (A) was founded on the strongest evidence (100% NJ/99% ML bootstrap support). Clade B comprised the entire population from Thailand and two samples from China (NGY5 and NGFA4), whereas the remainder of the Chinese samples clustered in a third clade (C). Bootstrap support for clades B and C was moderate (>80%), while within the Peninsular Malaysia and Thailand samples, evidence for population structure within each country was variable but sometimes exceeded 80%. However, although four distinct geographic sites had been sampled in Peninsular Malaysia, these subpopulations did not cluster strictly by location (Figure 4—note distribution of sample codes from Table 1). Phylogenetic trees constructed using individual gene markers produced similar tree topologies between COI (Figure S2) and the concatenated dataset but for ITS2, sequences from Malaysia and China were not clearly separated (Figure S3). The 18S rRNA gene exhibited the highest level of conservation between the three loci as expected, with only a single polymorphic site. This comprised two alleles, one in Thailand and one in China, which were observed together in Malaysia (Figure S4, Table S4).

### 3.3. Pairwise Distance and Species Delimitation Analysis

Pairwise intraspecific analysis of genetic distances for concatenated sequences of *N. gallinarum* ranged from zero to 3.55% (Table S1). The highest intraspecific divergence was recorded for an individual from BJV (KPGX18) compared with four individuals from BNAN at 3.55%, whereas the lowest divergence (zero) was seen between multiple individuals within the population from Peninsular Malaysia. At the country level, the pairwise genetic distance for concatenated genes between populations from Peninsular Malaysia and Thailand was 3.36%, whereas divergences of 2.64% and 2.36% separated the populations of Peninsular Malaysia and Thailand, respectively, from the Chinese populations. Maximum pairwise distances were considerably higher for COI (9.06%—Table S2) than for ITS2 (2.7%—Table S3). The species delimitation analyses conducted using ABGD, ASAP, and mPTP consistently identified three operational taxonomic units (OTUs). Notably, the ASAP analysis produced the lowest score of 2.00, while the mPTP analysis yielded the best multi-coalescent rate score of 112.25. The OTUs comprised (1) Peninsular Malaysia only (=clade A), (2) China minority clade + Thailand (=clade B), and (3) China majority clade (=clade C), as superimposed on the tree in Figure 4. In contrast, the molecular delimitations of GMYC revealed significant discrepancies, resulting in the identification of seven OTUs: three for Peninsular Malaysia (within clade A), one for Thailand (designated within clade B), and three for China (including two within clade B and one in clade C), as illustrated in Figure 4.

### 3.4. Haplotype Resolution and Network Analysis

**Figure 5:**
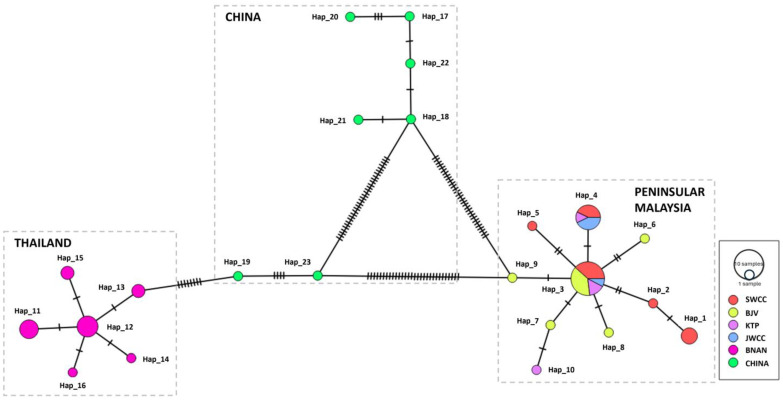
Minimum spanning haplotype network of *N. gallinarum* based on concatenated sequences isolated from four populations in Peninsular Malaysia, one population in Thailand, and the seven sequences from China obtained from Zhou et al. [30]. Each haplotype is represented by the coloured nodes and their relative sizes indicate haplotype frequency. Nodes of the same colour specify the haplotype from the same population. The dashed lines on each node connecting haplotypes represent polymorphisms.

Sixteen distinct haplotypes were recognised from the MSN constructed using the concatenated *N. gallinarum* gene datasets from Peninsular Malaysia and Thailand (*n* = 45), with a further seven originating from the published Chinese data (Figure 5). The MSN highlighted the unambiguous separation between the populations from Peninsular Malaysia and Thailand (zero haplotypes in common), and neither were any haplotypes shared with China. However, despite the Thailand specimens originating only from two villages within the same subdistrict, they were split into 6 haplotypes compared with 10 haplotypes found across the 4 subpopulations sampled in Peninsular Malaysia. Haplotype 3 was the most prevalent, including individuals from all four Peninsular Malaysia subpopulations (*n* = 13), followed by haplotype 4 found in three subpopulations (*n* = 7). Haplotypes 2, 5–10, 14, and 16 represented singletons (Table 3). Similarly, the MSN constructed using individual gene markers revealed no shared haplotype among the three examined countries for the COI gene (Figure S5, Table S5). However, in the case of ITS2 (comprising 12 haplotypes), populations from China demonstrated evidence of haplotype sharing with both Peninsular Malaysia and Thailand (Figure S6, Table S6). Finally, the 18S rRNA gene displayed just two haplotypes: Hap 1 was the only one present in Thailand and was a rare haplotype in Malaysia (restricted to Sungkai), whereas all Chinese and most Malaysian samples belonged to Hap 2 (Figure S7, Table S7).

### 3.5. Genetic Differentiation and Gene Flow

The AMOVA revealed that 96.41% of genetic variation was partitioned among groups of *N. gallinarum* from Peninsular Malaysia and Thailand (Table 4). The among-populations–within-groups variability (0.51%) was much lower than the genetic variation apparent within each population (3.08%). The variance component and fixation index were statistically significant for the among populations–within groups and within-population comparisons, but not for the among-groups analysis (Table 4).

The observed overall migrant per generation (N*m*) value of 0.02 and population subdivision (*F*_ST_) value of 0.933 indicated low gene flow that led to very high genetic differentiation among most populations of *N. gallinarum* studied (Table 5). The greatest *F*_ST_ value was observed in comparisons between each Peninsular Malaysia subpopulation and the population from Thailand (Table 5). However, the Mantel regression analysis showed no significant relationship between net *F*_ST_ and geographic distance among the five subpopulations of *N. gallinarum* (r = 0.962, *p* = 0.109) in Peninsular Malaysia and Thailand.

## 4. Discussion

The simplest definition of cryptic species is “two or more distinct species that are erroneously classified (and hidden) under one species name” [72,73]. However, a definition that takes account of the underlying biological processes involved in cryptic speciation would add that it is a low level of phenotypic distinctiveness coupled with clear genetic differentiation that exemplifies cryptic species [73]. Evidence for cryptic speciation has been uncovered across the diversity of life and, in 2015, a review of cryptic species in Acari found that the phenomenon had been reported from 24 of the 142 acarine superfamilies, although the greatest predictor of cryptic species discovery was the research effort expended on specific taxa [74]. In the current study, populations of *N. gallinarum* from two countries (Malaysia and Thailand) exhibited similar features based on morpho-taxonomic identification, differing significantly only in the length of the legs. Minor morphological features alone are often unreliable for the accurate identification of sibling or cryptic species [75] and in *N. gallinarum*, the lack of marked morphological differences contrasted with deep splits in concatenated molecular markers between chiggers originating from Thailand and Peninsular Malaysia. Moreover, most of the published sequences from southeastern China formed a third, separated clade.

Multi-locus sequence analysis studies have increased in popularity over the years due to the reduced impact of evolutionary rates for individual genes [76,77]; for instance, several such studies have been performed in ticks, usually using concatenated mitochondrial markers [78,79,80,81]. While mitochondrial DNA undergoes a more rapid rate of mutation compared to nuclear DNA [82] and recombination in animal mitogenomes is considered rare [83], its utility in identifying distinct maternal lineages is counterbalanced by caveats when applied to the detection of reproductive isolation [84]. Hence, combining nuclear and mitochondrial loci as performed here is favourable for population genetic analyses.

Our study revealed a genetic divergence of 3.36% between Peninsular Malaysia and Thailand, revealing the potential existence of a species complex and reminiscent of recent studies in the region on *Simulium* spp. blackflies [85,86,87]. According to Pramual et al. [88], a divergence of >3% indicates a substantiated threshold signifying distinct separation between sister phylogroups. Notably, the ABGD, ASAP, and mPTP methods concorded in delineating the Peninsular Malaysia and Thailand specimens into two separate OTUs, and the MSN analysis showed a lack of shared concatenated haplotypes between them. However, although a proportion of the published data from southeastern China were classified in the same OTU as the Thailand specimens with most species delimitation methods, the MSN analysis demonstrated that none of the concatenated haplotypes reported from southeastern China were shared with Peninsular Malaysia or Thailand. This finding is more consistent with the GMYC analysis, but we propose that a conservative approach be taken with respect to the potential numbers of cryptic species until more data are available, especially from the Chinese populations. Despite being collected from just two villages within the same subdistrict, the Thailand specimens exhibited 6 distinct haplotypes, whereas only 10 haplotypes were found across the 4 subpopulations sampled in Peninsular Malaysia. Koopman et al. [89] proposed that the presence of shared haplotypes among different subpopulations indicates recent gene flow in the population, as seen with the specimens from Peninsular Malaysia. Haplotype 3 within the population from Peninsular Malaysia was the most prevalent and may represent the ancestral haplotype due to its representation in a significant proportion of individuals across all subpopulations and its centralised placement in the network [90]. Moreover, Hap 3 may also be a stable haplotype with diverse environmental adaptability [91].

Clearly, the COI gene provided the greatest resolution among the specimens analysed here with no haplotypes in common between countries, whereas both ITS2 and 18S exhibited shared haplotypes in two of the three countries. To the best of our knowledge, only one other analysis of the ITS2 region in chiggers has been published, and this found no evidence of intraspecific variation in the genera *Leptotrombidium*, *Neotrombicula*, and *Euschoengastia* in South Korea [32], although the geographic extent of sampling was very limited. Regarding the application of 18S rRNA sequencing in chiggers, it has been used for confirmation of species identification in studies from Brazil [92] and South Korea [93], in which the gene was found to be invariant within species. Thus, the identification here of several haplotypes for ITS2 and 18S provides corroborating evidence for cryptic speciation in *N. gallinarum* independently of the COI mitochondrial marker, even if shared haplotypes between countries are present at the nuclear level.

Greater genetic differentiation among populations can hinder gene flow [94]. This phenomenon was observed in our study, with high separation between the two sampled nations, and total interpopulation gene flow was limited (N*m* = 0.02) by the increase in geographical distance. This genetic divergence may underlie the species’ adaptability to their specific geographical habitat and local environmental changes across the national border. Recently, Tao et al. [39] published a population genetic study of *N. gallinarum* in four provinces of China with a larger sample size (*n* = 192) than we achieved here. Unfortunately, a direct comparison with their study was not possible, as we were unable to amplify the ND5 locus of *N. gallinarum* used by these workers. They found that *N. gallinarum* in southern China was divided into two clades, but there was little evidence of genetic isolation between geographic sites. One exception was the population from Jiangxi, which displayed limited gene flow with *N. gallinarum* from other provinces, although it was still much greater than that between Thailand and Peninsular Malaysia. Generally, high gene flow with low to moderate genetic differentiation was observed between the subpopulations of southern China, while genetic variation within the population as a whole was higher than that among subpopulations, which is in accordance with our findings in Peninsular Malaysia. In China, trade in commercial lines of poultry between provinces may have facilitated gene flow in *N. gallinarum*, as chiggers have very limited intrinsic dispersal ability. Conversely, in Peninsular Malaysia and Thailand, traditional rearing of local chicken breeds at the village level is likely to drive reproductive isolation in parasites of poultry.

In sexual populations, increases in gene flow will lead to an increase in genetic diversity. In general, homogeneous environments contribute to reduced levels of genetic diversity, while heterogeneous environments, including variations in geography, climate, vegetation, and other factors, result in higher levels of genetic diversity [91,95]. The collection of *N. gallinarum* from both domestic and wild birds in various habitats in Peninsular Malaysia (e.g., forests, sanctuaries, and villages) may have contributed to greater genetic diversity in comparison to only one ecotype (villages) from Thailand, but broader sampling in Thailand will be required to unravel the potential impacts of environmental and host factors. In Peninsular Malaysia, *N. gallinarum* infested a wide range of bird host species, with *L. rufa* (Malayan crested fireback) and *P. inopinatum* (Mountain peacock-pheasant) noted as new host records [29] for this chigger species. Due to their decreasing population trends, *L. rufa*, *P. inopinatum*, and *P. malacense* (Malayan peacock-pheasant) are categorised as totally protected species in Malaysia [96] and classed as either “vulnerable” (*L. rufa*, *P. inopinatum*) or “endangered” (*P. malacense*) by the International Union for Conservation of Nature [97,98,99,100]. The Department of Wildlife and National Parks of Peninsular Malaysia is proactively involved in searching for these species within their native habitats. Any individuals located may be captured and subsequently placed in captivity for the specific intention of breeding [101]. This breeding program could introduce chigger mites into the captive environment, and this may explain the gene exchange between the population from KTP (forest) and those from JWCC and SWCC (captive breeding sanctuaries). Strong selection by host in *N. gallinarum* appears to be unlikely, as Hap 3 and 4 were recovered from several different bird species.

A previous study on a chigger species from Poland [*Hirsutiella zachvatkini* (Schluger, 1948)] revealed host-dependent morphological plasticity in the leg, but not scutal, characters in the absence of differentiation based on COI barcodes [102]. However, in other species from Poland and Greece, such as *Leptotrombidium europaeum* (Daniel and Brelih, 1959) and *Neotrombicula talmiensis* Schluger, 1955, respectively, high diversity in morphology was observed within a single OTU, while some congeneric specimens were morphologically similar to these two species but were assigned to different OTUs by ABGD analysis of COI sequences [35]. Beyond Europe, substantial intraspecific diversity of COI has been reported within *Leptotrombidium* spp. in Southeast Asia [31], South Korea [32], and Japan [34], and for *Walchia* spp. in Southeast Asia [31,103], sometimes even in chiggers of the same species collected from a single host. However, as chigger populations can harbour several vertically transmitted bacteria with the potential to induce reproductive manipulations [3] and cytonuclear discordance [104], it is important to investigate potential cryptic species using nuclear as well as mitochondrial loci as we have explored here.

## 5. Conclusions

The use of multi-locus sequence analysis of both mitochondrial-encoded and nuclear-encoded genes revealed that *N. gallinarum* populations in Peninsular Malaysia and Thailand are geographically isolated with restricted gene flow leading to unambiguous genetic differentiation. High genetic diversity was attributed to the population in Peninsular Malaysia; however, more exploration is needed to elucidate the genetic diversity of *N. gallinarum* in Thailand, which was high even in two adjacent villages within the same subdistrict. Finally, our study revealed three robustly supported genetic lineages in Asia and further denoted *N. gallinarum* as a potential species complex, although further studies are required to determine the extent of biological differences (including pathogenicity) between its members.

## Figures and Tables

**Table 1:** Information on geographical origin and host species of *N. gallinarum*.

Country	Population Code	Locality	Coordinates	Habitat Type	Host Species	Chigger ID	GenBank Accession No.		
							COX1	ITS2	18S
Peninsular Malaysia	SWCC	Sungkai Wildlife Conservation Centre, Perak	E101.36623, N4.06430	Sanctuary	*Lophura rufa*	SWX3	OR632279	OR636401	OR632359
					*Polyplectron inopinatum*	SWX10	OR632280	OR636402	OR632360
					*P. inopinatum*	SWX12	OR632281	OR636403	OR632361
					*P. inopinatum*	SWX13	OR632282	OR636404	OR632362
					*P. inopinatum*	SWX14	OR632283	OR636405	OR632363
					*P. inopinatum*	SWX15	OR632284	OR636406	OR632364
					*P. inopinatum*	SWX19	OR632285	OR636407	OR632365
					*P. inopinatum*	SWX21	OR632286	OR636408	OR632366
					*P. inopinatum*	SWX22	OR632287	OR636409	OR632367
					*P. inopinatum*	SWX23	OR632288	OR636410	OR632368
					*P. inopinatum*	SWX37	OR632289	OR636411	OR632369
					*P. inopinatum*	SWX40	OR632290	OR636412	OR632370
					*L. rufa*	SWX43	OR632291	OR636413	OR632371
	BJV	Bestari Jaya Village, Selangor	E101.41022, N3.37801	Village	*Gallus gallus domesticus*	KPGX5	OR632292	OR636414	OR632372
					*G. gallus domesticus*	KPGX6	OR632293	OR636415	OR632373
					*G. gallus domesticus*	KPGX9	OR632294	OR636416	OR632374
					*G. gallus domesticus*	KPGX10	OR632295	OR636417	OR632375
					*G. gallus domesticus*	KPGX11	OR632296	OR636418	OR632376
					*G. gallus domesticus*	KPGX18	OR632297	OR636419	OR632377
					*G. gallus domesticus*	KPGX19	OR632298	OR636420	OR632378
					*G. gallus domesticus*	KPGX20	OR632299	OR636421	OR632379
					*G. gallus domesticus*	KPGX21	OR632300	OR636422	OR632380
	JWCC	Jemaluang Wildlife Conservation Centre, Johor	E103.85297, N2.29136	Sanctuary	*L. rufa*	JWX3	OR632301	OR636423	OR632381
					*Polyplectron malacense*	JWX17	OR632302	OR636424	OR632382
					*P. malacense*	JWX18	OR632303	OR636425	OR632383
					*P. malacense*	JWX20	OR632304	OR636426	OR632384
	KTP	Kota Tinggi Plantation, Johor	E103.86604, N2.03023	Forest	*G. gallus*	KTX7	OR632305	OR636427	OR632385
					*G. gallus*	KTX8	OR632306	OR636428	OR632386
					*G. gallus*	UMPX2	OR632307	OR636429	OR632387
					*G. gallus*	CHX2	OR632308	OR636430	OR632388
Thailand	BNAN	Ban Huay Muang and Ban Santisuk, Saen Thong subdistrict, Tha Wang Pha, Nan	E100.71897, N19.13999; E100.69891, N19.12957	Village	*G. gallus domesticus*	BNANX2	OR632309	OR636431	OR632389
					*G. gallus domesticus*	BNANX8	OR632310	OR636432	OR632390
					*G. gallus domesticus*	BNANX9	OR632311	OR636433	OR632391
					*G. gallus domesticus*	BNANX10	OR632312	OR636434	OR632392
					*G. gallus domesticus*	BNANX11	OR632313	OR636435	OR632393
					*G. gallus domesticus*	BNANX12	OR632314	OR636436	OR632394
					*G. gallus domesticus*	BNANX13	OR632315	OR636437	OR632395
					*G. gallus domesticus*	BNANX14	OR632316	OR636438	OR632396
					*G. gallus domesticus*	BNANX15	OR632317	OR636439	OR632397
					*G. gallus domesticus*	BNANX16	OR632318	OR636440	OR632398
					*G. gallus domesticus*	BNANX17	OR632319	OR636441	OR632399
					*G. gallus domesticus*	BNANX19	OR632320	OR636442	OR632400
					*G. gallus domesticus*	BNANX20	OR632321	OR636443	OR632401
					*G. gallus domesticus*	BNANX21	OR632322	OR636444	OR632402
					*G. gallus domesticus*	BNANX22	OR632323	OR636445	OR632403

**Table 2:** Diagnosis and morphometry comparisons of *N. gallinarum* voucher specimens from Peninsular Malaysia and Thailand.

Morphometry Measurements (µm)												
	AW	PW	SB	ASB	PSB	AP	AM	AL	PL	S	H	IP
Peninsular Malaysia												
*n* = 11												
Mean	52	67	42	21	25	28	30	43	39	26	43	686
Min	48	64	39	17	24	27	25	38	36	23	39	625
Max	60	74	44	25	30	31	34	48	46	31	49	704
Thailand												
*n* = 7												
Mean	52	69	43	21	25	30	28	44	42	24	43	713
Min	49	63	41	19	24	28	25	41	38	18	39	701
Max	53	74	45	23	27	31	32	48	46	34	46	726
Mann–Whitney U-test												
*U*	36.000	28.500	30.500	37.000	36.000	17.000	27.000	32.000	22.000	19.000	33.500	1.500
*Z*	−0.236	−0.924	−0.748	−0.139	−0.235	−1.993	−1.049	−0.596	−1.507	−1.805	−0.457	−3.361
*P*	0.860	0.375	0.479	0.930	0.860	0.056	0.328	0.596	0.151	0.085	0.659	<0.001 *

Note: Statistical analysis was performed with exact significance using SPSS software v. 26. AW—distance between anterolateral setae; PW—distance between posterolateral setae; SB—distance between sensilla bases; ASB—distance between sensillary bases line and anterior margin of scutum; PSB—distance between sensillary bases line and posterior margin of scutum; AP—distance between anterolateral setae and posterolateral setae; AM—length of anteromedial setae; AL—length of anterolateral setae; PL—length of posterolateral setae; S—length of scutal sensilla; H—length of humeral setae; IP—total length of leg. * Asterisk indicates the parameter with a significant statistical test (*p* < 0.05).

**Table 3:** Haplotype (hap) frequency of five populations of *N. gallinarum* in Malaysia and Thailand by region.

Hap	*N. gallinarum* Individuals from Each Study Region (*n*)				
	Peninsular Malaysia				Thailand
	SWCC (13)	BJV (9)	JWCC (4)	KTP (4)	BNAN (15)
1	3	0	0	0	0
2	1	0	0	0	0
3	5	5	1	2	0
4	3	0	3	1	0
5	1	0	0	0	0
6	0	1	0	0	0
7	0	1	0	0	0
8	0	1	0	0	0
9	0	1	0	0	0
10	0	0	0	1	0
11	0	0	0	0	4
12	0	0	0	0	5
13	0	0	0	0	2
14	0	0	0	0	1
15	0	0	0	0	2
16	0	0	0	0	1
Total hap	5	5	2	3	6

Note. SWCC: Sungkai Wildlife Conservation Centre; BJV: Bestari Jaya Village; JWCC: Jemaluang Wildlife Conservation Centre; KTP: Kota Tinggi Plantation; BNAN: Tha Wang Pha, Nan Province.

**Table 4:** Measures of geographical population differentiation in *N. gallinarum* based on AMOVA.

Source of Variation	d.f.	Sum of Square	Variance Components	Variation (%)	Fixation Index (Φ)	Significance Test (*p*)
Among groups	1	442.689	22.02220	96.41	0.96407	0.197
Among populations within groups	3	4.528	0.11737	0.51	0.14299	0.031 *
Within population	40	28.138	0.70346	3.08	0.96920	0.00 *

Note: * Significant *p* < 0.05.

**Table 5:** Number of migrants per generation (N*m*) and population subdivision (*F*_ST_) of *N. gallinarum* in relation to the geographical distance.

Populations		Distance (km)	Migrant per Generation (N*m*)	Population Subdivision (*F*_ST_)
SWCC	BJV	108	1.51	0.14189
SWCC	JWCC	499	0.72	0.25882
SWCC	KTP	525	2.78	0.08247
SWCC	BNAN	2081	0.01	0.96553
BJV	JWCC	421	0.46	0.35294
BJV	KTP	437	−6.38	−0.04082
BJV	BNAN	2166	0.01	0.97279
JWCC	KTP	33	2.00	0.11111
JWCC	BNAN	2552	0.00	0.98168
KTP	BNAN	2598	0.01	0.97093
Whole population			0.02	0.93312

Note. SWCC: Sungkai Wildlife Conservation Centre; BJV: Bestari Jaya Village; JWCC: Jemaluang Wildlife Conservation Centre; KTP: Kota Tinggi Plantation; BNAN: Tha Wang Pha, Nan Province.

## Data Availability

Sequences generated were deposited in the GenBank database (https://www.ncbi.nlm.nih.gov/GenBank, accessed 4 October 2023) under the accession numbers OR632279-OR632323 for the COI gene, OR636401-OR636445 for the ITS2 gene, and OR632359-OR632403 for the 18S rRNA gene.

## Supplementary Materials

The following supporting information can be downloaded at https://www.mdpi.com/article/10.3390/ani14060980/s1, Figure S1: Phylogenetic relationships among *N. gallinarum* populations from Peninsular Malaysia, Thailand, and China were inferred through Bayesian Inference analysis based on the concatenated nucleotide sequences of mitochondrial cytochrome c oxidase subunit 1, second internal transcribed spacer, and 18S ribosomal DNA. Vertical bars on the right are the population groups. Coloured branches indicate different countries: red for samples from Peninsular Malaysia, green for samples from Thailand, and blue for samples from China. Figure S2: Phylogenetic relationships among *N. gallinarum* populations from Peninsular Malaysia, Thailand, and China were inferred through Neighbour Joining (NJ) and Maximum Likelihood (ML) analysis based on the sequences of mitochondrial cytochrome c oxidase subunit 1 (COI). Bootstrap values (NJ/ML) are shown on the branches. Vertical bars on the right are the population groups. Coloured branches indicate different countries: red for samples from Peninsular Malaysia, green for samples from Thailand, and blue for samples from China. Bootstrap values less than 0.50 are not shown in the figure. Figure S3: Phylogenetic relationships among *N. gallinarum* populations from Peninsular Malaysia, Thailand, and China were inferred through Neighbour Joining (NJ) and Maximum Likelihood (ML) analysis based on the sequences of nuclear-encoded internal transcribed spacer 2 (ITS2). Bootstrap values (NJ/ML) are shown on the branches. Coloured branches indicate different countries: red for samples from Peninsular Malaysia, green for samples from Thailand, and blue for samples from China. Bootstrap values less than 0.50 are not shown in the figure. Figure S4: Phylogenetic relationships among *N. gallinarum* populations from Peninsular Malaysia, Thailand, and China were inferred through Neighbour Joining (NJ) and Maximum Likelihood (ML) analysis based on the sequences of nuclear-encoded 18S. Bootstrap values (NJ/ML) are shown on the branches. Vertical bars on the right are the population groups. Coloured branches indicate different countries, red for samples from Peninsular Malaysia, green for samples from Thailand, and blue for samples from China. Bootstrap values less than 0.50 are not shown in the figure. Figure S5: Minimum spanning haplotype network of *N. gallinarum* based on COI gene sequence isolated from four populations in Peninsular Malaysia, one population in Thailand, and the seven sequences from China obtained from Zhou et al. [30]. Each haplotype is represented by the coloured nodes and their relative sizes indicate haplotype frequency. Nodes of the same colour specify the haplotype from the same population. The dashed lines on each node connecting haplotypes represent polymorphisms. Figure S6: Minimum spanning haplotype network of *N. gallinarum* based on the ITS2 gene sequence isolated from four populations in Peninsular Malaysia, one population in Thailand, and the seven sequences from China obtained from Zhou et al. [30]. Each haplotype is represented by the coloured nodes and their relative sizes indicate haplotype frequency. Nodes of the same colour specify the haplotype from the same population. The dashed lines on each node connecting haplotypes represent polymorphisms. Figure S7: Minimum spanning haplotype network of *N. gallinarum* based on the 18S ribosomal DNA isolated from four populations in Peninsular Malaysia, one population in Thailand, and the seven sequences from China obtained from Zhou et al. [30]. Each haplotype is represented by the coloured nodes and their relative sizes indicate haplotype frequency. Nodes of the same colour specify the haplotype from the same population. The dashed lines on each node connecting haplotypes represent polymorphisms. Table S1: Pairwise genetic distance based on concatenated genes between populations from Peninsular Malaysia, Thailand, and China computed using the Kimura 2-parameter (K2P) model from MEGA11. Table S2: Pairwise genetic distance for the COI gene between populations from Peninsular Malaysia, Thailand, and China computed using the Kimura 2-parameter (K2P) model from MEGA11. Table S3: Pairwise genetic distance for the ITS2 gene between populations from Peninsular Malaysia, Thailand, and China computed using the Kimura 2-parameter (K2P) model from MEGA11. Table S4: Pairwise genetic distance for the 18S rRNA gene between populations from Peninsular Malaysia, Thailand, and China computed using the Kimura 2-parameter (K2P) model from MEGA11. Table S5: Haplotype frequency of five populations of *N. gallinarum* in Malaysia and Thailand by region based on the COI gene. Table S6. Haplotype frequency of five populations of *N. gallinarum* in Malaysia and Thailand by region based on the ITS2 gene. Table S7: Haplotype frequency of five populations of *N. gallinarum* in Malaysia and Thailand by region based on the 18S rRNA gene.

## Abbreviations

ASAP: Assemble species by automatic partitioning; ABGD: Automatic barcode gap discovery; CIPRES: Cyber Infrastructure for Phylogenetic Research; COI: Cytochrome c oxidase subunit I; GMYC: Generalised Mixed Yule Coalescent; ITS2: Internal transcribed spacer 2; mPTP: multi-rate Poisson Tree Processes; PCR: Polymerase chain reaction; OTU: Operational taxonomic unit.
