# Supplementary material for: Multi-Locus Sequence Analysis Indicates Potential Cryptic Speciation in the Chigger Mite Neoschoengastia gallinarum (Hatori, 1920) Parasitising Birds in Asia
Source: Animals (Basel). 2024 Mar 21;14(6):980. doi: 10.3390/ani14060980 (PMC10967278; doi:10.3390/ani14060980)
Supplement: Supplementary file 1 [file animals-14-00980-s001.zip › Additional File S1.pdf]

Supplementary information

Figure S1

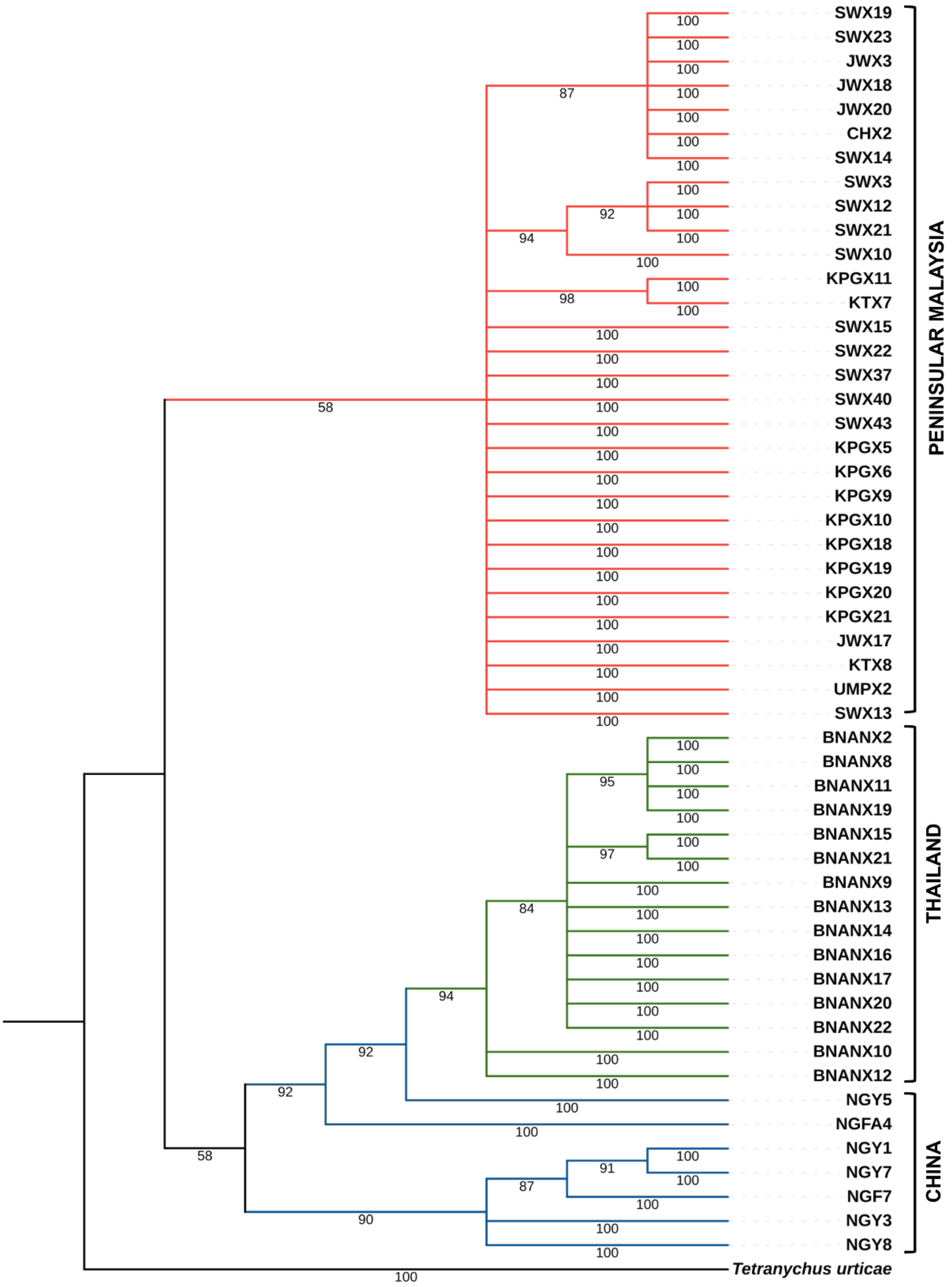

Phylogenetic relationships among *N. gallinarum* populations from Peninsular Malaysia, Thailand, and China were inferred through Bayesian Inference analysis based on the concatenated nucleotide sequences of mitochondrial cytochrome c oxidase subunit 1, second internal transcribed spacer, and 18S ribosomal DNA. Vertical bars on the right are the population groups. Coloured branches indicate different countries: red for samples from Peninsular Malaysia, green for samples from Thailand, and blue for samples from China.

Figure S2

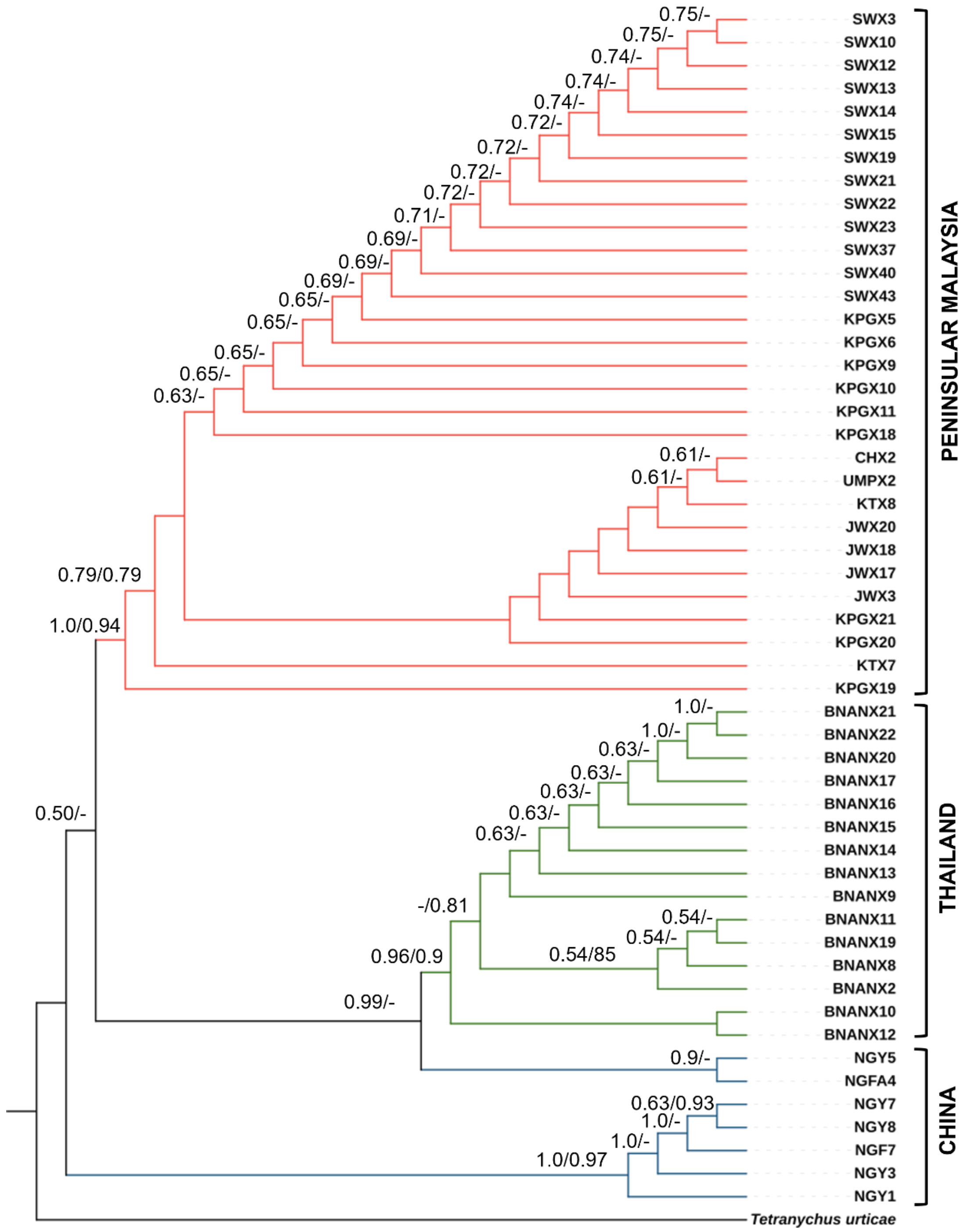

Phylogenetic relationships among *N. gallinarum* populations from Peninsular Malaysia, Thailand, and China were inferred through Neighbour Joining (NJ) and Maximum Likelihood (ML) analysis based on the sequences of mitochondrial cytochrome c oxidase subunit 1 (COI). Bootstrap values (NJ/ML) are shown on the branches. Vertical bars on the right are the population groups. Coloured branches indicate different countries, red for samples from Peninsular Malaysia, green for samples from Thailand and blue for samples from China. Bootstrap values less than 0.50 are not shown in the figure.

Figure S3

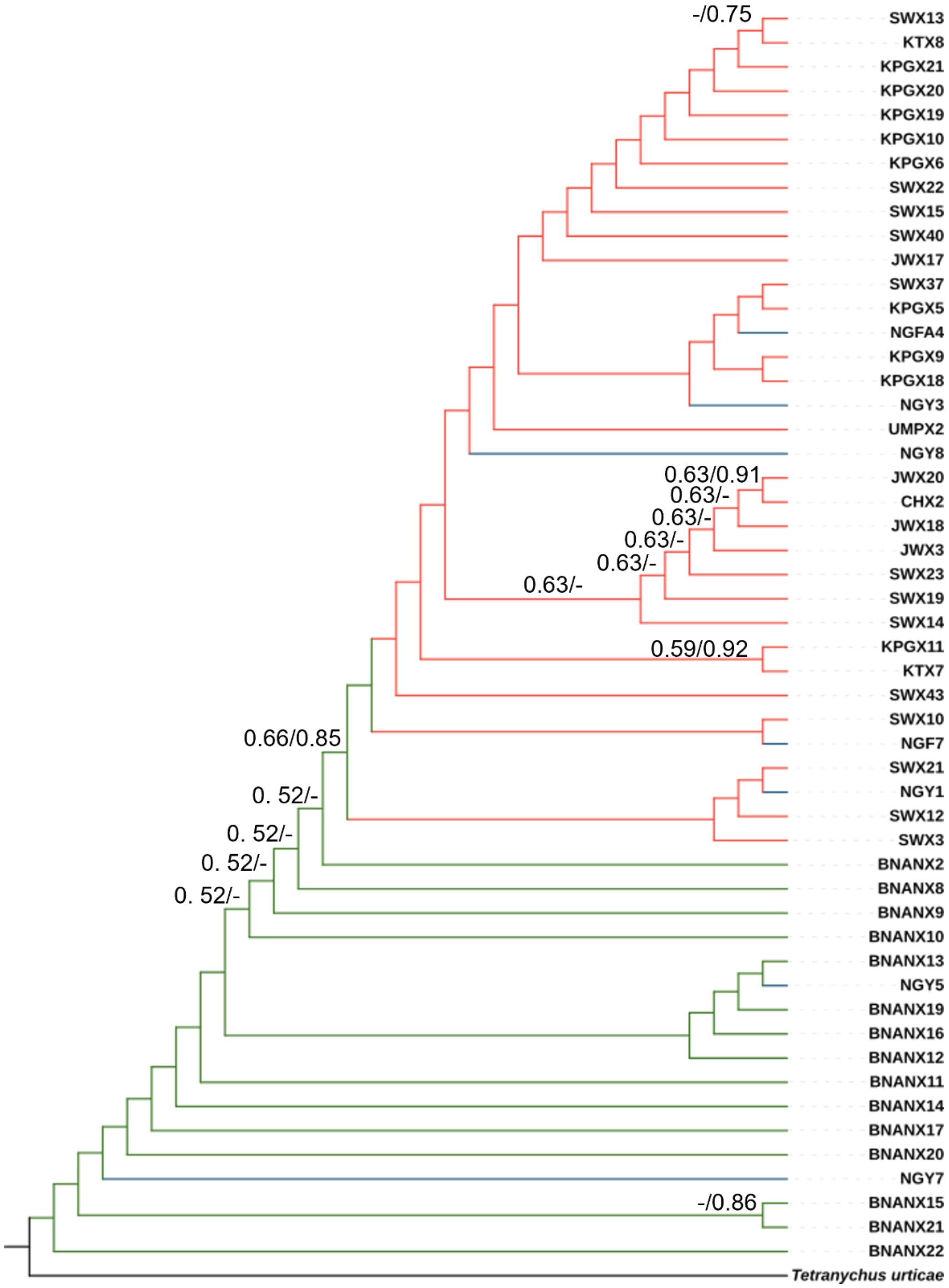

Phylogenetic relationships among *N. gallinarum* populations from Peninsular Malaysia, Thailand, and China were inferred through Neighbour Joining (NJ) and Maximum Likelihood (ML) analysis based on the sequences of nuclear-encoded internal transcribed spacer 2 (ITS2). Bootstrap values (NJ/ML) are shown on the branches. Coloured branches indicate different countries, red for samples from Peninsular Malaysia, green for samples from Thailand and blue for samples from China. Bootstrap values less than 0.50 are not shown in the figure.

Figure S4

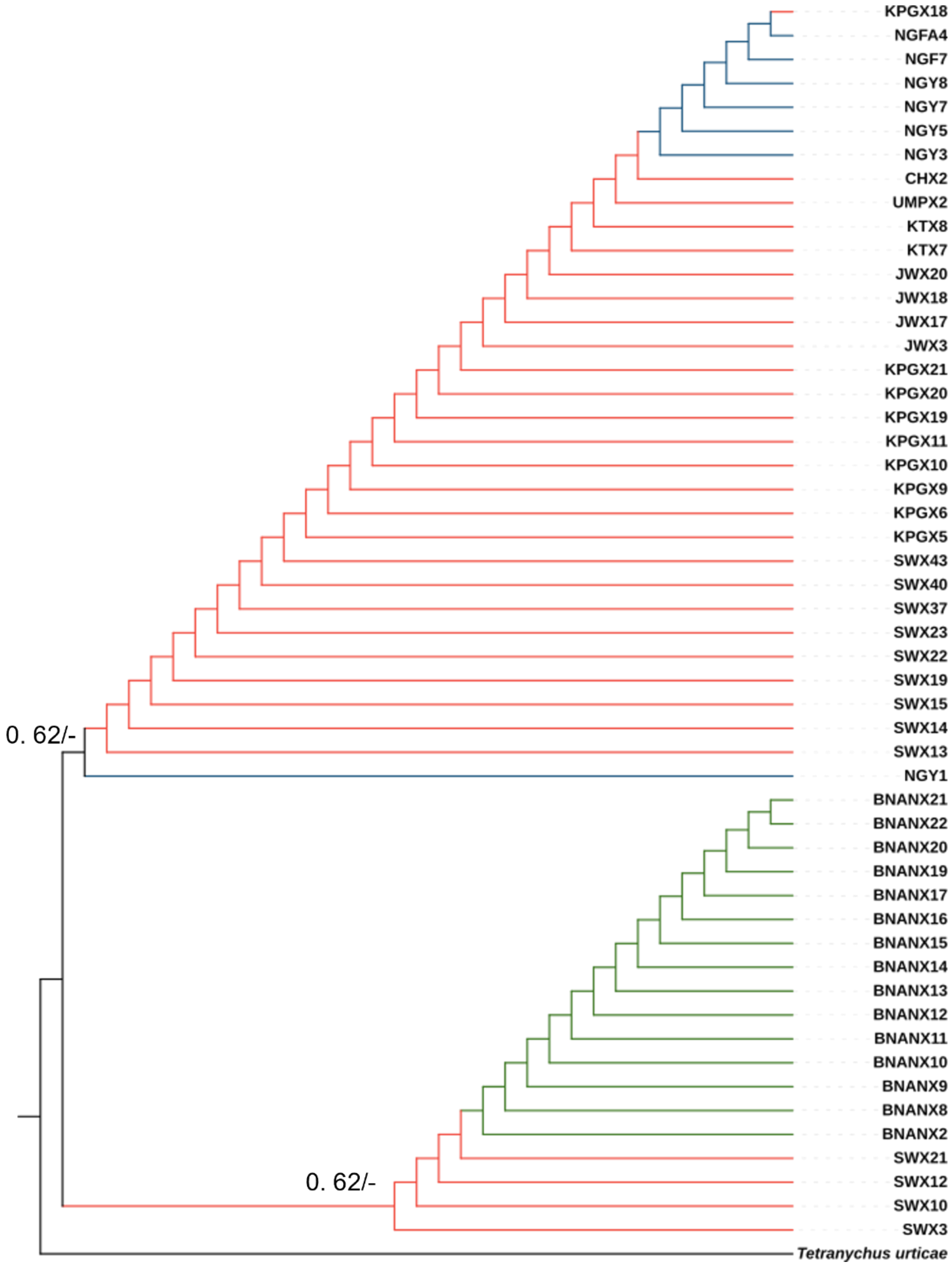

Phylogenetic relationships among *N. gallinarum* populations from Peninsular Malaysia, Thailand, and China were inferred through Neighbour Joining (NJ) and Maximum Likelihood (ML) analysis based on the sequences of nuclear-encoded 18S. Bootstrap values (NJ/ML) are shown on the branches. Vertical bars on the right are the population groups. Coloured branches indicate different countries, red for samples from Peninsular Malaysia, green for samples from Thailand and blue for samples from China. Bootstrap values less than 0.50 are not shown in the figure.

Figure S5

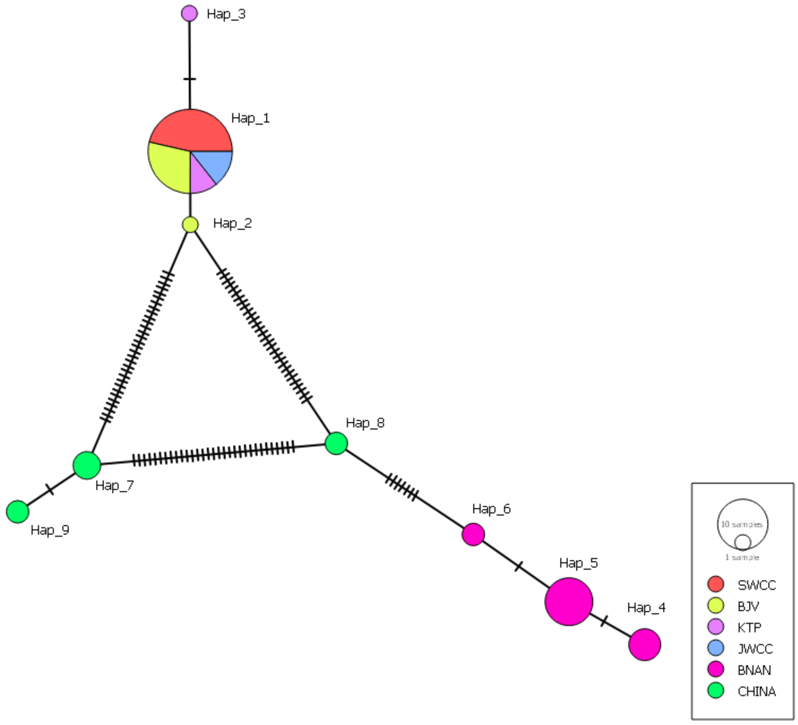

Minimum spanning haplotype network of *N. gallinarum* based on the mitochondrial COI gene isolated from four populations in Peninsular Malaysia, one population in Thailand, and the seven sequences from China obtained from Zhou *et al*. [30]. Each haplotype is represented by the coloured nodes and their relative sizes indicate haplotype frequency. Nodes of the same colour specify the haplotype from the same population. The dashed lines on each node connecting haplotypes represent polymorphisms.

Figure S6

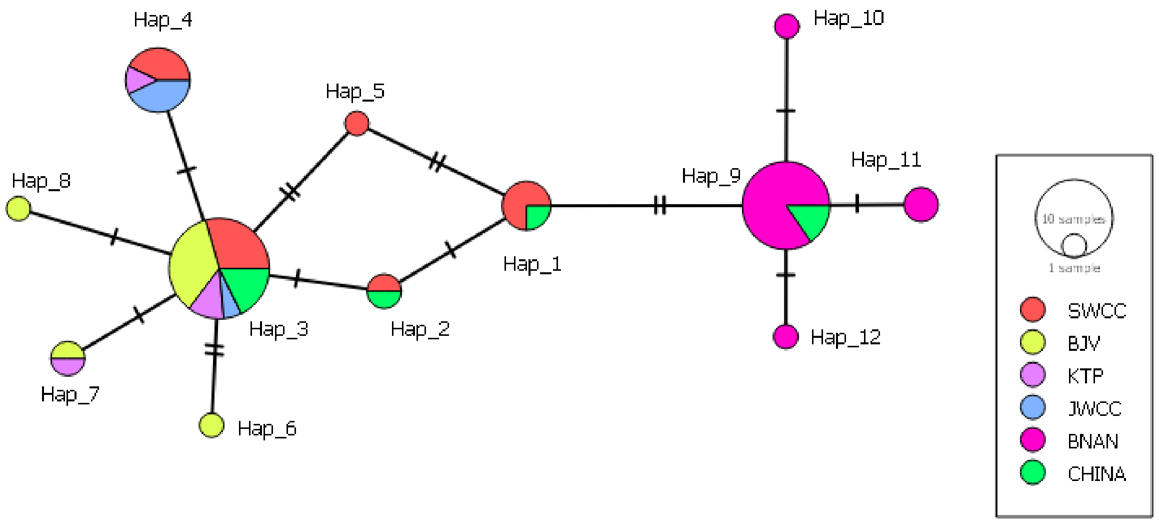

Minimum spanning haplotype network of *N. gallinarum* based on the ITS2 gene isolated from four populations in Peninsular Malaysia, one population in Thailand, and the seven sequences from China obtained from Zhou *et al.* [30]. Each haplotype is represented by the coloured nodes and their relative sizes indicate haplotype frequency. Nodes of the same colour specify the haplotype from the same population. The dashed lines on each node connecting haplotypes represent polymorphisms.

Figure S7

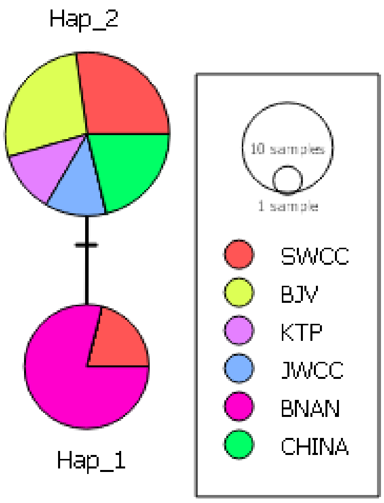

Minimum spanning haplotype network of *N. gallinarum* based on the 18S ribosomal DNA isolated from four populations in Peninsular Malaysia, one population in Thailand, and the seven sequences from China obtained from Zhou *et al.* [30]. Each haplotype is represented by the coloured nodes and their relative sizes indicate haplotype frequency. Nodes of the same colour specify the haplotype from the same population. The dashed lines on each node connecting haplotypes represent polymorphisms.
